# Real-World Comparison of Trough- and Online-Bayesian-Calculator-Derived AUC_0–24_/MIC Target Attainment for Vancomycin Monitoring in Critically Ill Patients

**DOI:** 10.3390/ph19071109

**Published:** 2026-07-18

**Authors:** Sufyan Alomair, Zahra Alsultan, Fatimah Alsultan, Fatimah Alghadeer, Alzahraa Aljafar, Anas Alkhawaldeh, Ayat Alherz, Batool Alhassan

**Affiliations:** 1 Department of Pharmacy Practice, College of Clinical Pharmacy, King Faisal University, Al-Ahsaa 31982, Saudi Arabia; zahraa.alsultan73@gmail.com (Z.A.); drfatimaalsultan@gmail.com (F.A.); fatima.17ghg@gmail.com (F.A.); alzahra.aljafar4@gmail.com (A.A.); 2 Almoosa Specialist Hospital, Al-Ahsaa 31982, Saudi Arabia

**Keywords:** vancomycin, AUC_0–24_/MIC, trough, discordance, critically ill, ClinCalc

## Abstract

**Introduction:** The preferred PK/PD goal for vancomycin is now AUC_0–24_/MIC-guided dosing, which requires two blood samples, additional time, increased cost, and possibly specialized staff, potentially limiting its use. Our study compared vancomycin target attainment using trough versus single-sample Bayesian AUC_0–24_/MIC in critically ill patients, using a validated online calculator. **Methods:** This retrospective cohort study included adults aged ≥18 years with stable renal function who were receiving vancomycin. Exclusions were age under 18, unstable renal function, hemodialysis, missing data, or non-steady-state vancomycin. Steady-state troughs were obtained, and AUC_0–24_/MIC was calculated using ClinCalc. The primary endpoint was discordance in target attainment between trough (15–20 mg/L) and AUC_0–24_/MIC (400–600 mg·h/L). The secondary endpoint was AKI within 48 h of initiating vancomycin. **Results:** The mean trough was 14.8 ± 8.0 mg/L, and the mean AUC_0–24_/MIC was 499.7 ± 201.3 mg·h/L. Trough levels were within target in 20%, and AUC_0–24_/MIC in 67%. The 3 × 3 cross-tabulation showed a significant association (χ^2^ = 27.33, *p* < 0.001), but only fair agreement (Cohen’s κ = 0.215) and a biased disagreement pattern (*p* < 0.001). Thirty-eight patients (38%) had “false alarming” results: trough < 15 mg/L despite AUC_0–24_/MIC ≥ 400 mg·h/L. Three patients (3.0%) showed “falsely reassuring” results: trough 15–20 mg/L with AUC_0–24_/MIC > 600 mg·h/L. The odds of supratherapeutic AUC_0–24_/MIC > 600 mg·h/L were tenfold higher in patients with trough 15–20 mg/L than in those below 15 mg/L (OR 10.24, *p* = 0.048). **Conclusions:** In critically ill ICU patients, calculator-derived AUC_0–24_/MIC showed significant discordance with single-trough vancomycin monitoring, primarily indicating apparent under-exposure by trough criteria in patients with adequate AUC_0–24_/MIC levels, which may lead to unnecessary dose escalation.

## 1. Introduction

Vancomycin remains the first-line antibiotic for serious infections caused by methicillin-resistant *Staphylococcus aureus* (MRSA) [1]. Optimal therapeutic drug monitoring is crucial because of the drug’s narrow therapeutic window; subtherapeutic levels can lead to treatment failure, and supratherapeutic levels may result in nephrotoxicity [2]. Vancomycin therapy has traditionally been guided by trough-based monitoring because it is a useful and straightforward technique. However, this approach has serious disadvantages, including the need for precise sampling at the appropriate time and a weak correlation with overall drug exposure. The 2009 vancomycin consensus guideline endorsed a steady-state trough concentration of 15–20 mg/L as a surrogate for the area under the concentration–time curve to minimum inhibitory concentration ratio (AUC_0–24_/MIC) of ≥400 mg·h/L, reasoning that troughs of this magnitude would generally correspond to adequate AUC_0–24_/MIC values when the assumed MIC is 1 mg/L [3].

However, several pharmacokinetic studies have challenged this assumption. Multiple studies have demonstrated that trough levels underestimate the true AUC_0–24_/MIC by approximately 25%, that troughs in the 10–20 mg/L range can correspond to a wide range of AUC_0–24_/MIC values, and that targeting troughs ≥ 15 mg/L often results in supratherapeutic AUC_0–24_/MIC exposure and an elevated risk of nephrotoxicity [4,5,6]. The 2020 updated ASHP/IDSA/PIDS/SIDP consensus guideline recommends an AUC_0–24_/MIC of 400–600 mg·h/L as the optimal pharmacodynamic target and advises against relying solely on trough levels [2].

Implementing AUC_0–24_/MIC-based dosing remains challenging in many institutions. This is due to several factors, including the need for two blood samples (peak and trough), higher costs, increased nurse workload, and the requirement for specialized software and trained personnel, which make implementation difficult, particularly in high-demand settings with a large number of patients [7]. Validated commercial Bayesian dose-optimization platforms can replace traditional AUC_0–24_/MIC calculations to estimate vancomycin dosing regimens in adult patients. ClinCalc, in particular, offers both Bayesian and first-order analytic calculation modes free of charge, allowing single-level estimation when only one trough is available [8].

However, to our knowledge, the agreement between single-level calculator-derived AUC_0–24_/MIC and trough-only monitoring has not been evaluated in critically ill patients, in whom critical illness-related disturbances in pharmacokinetic profiles can distort vancomycin pharmacokinetics. Therefore, the study aims to compare target attainment of vancomycin trough concentrations and ClinCalc-derived AUC_0–24_/MIC in critically ill patients using Bayesian single-level estimation.

## 2. Results

### 2.1. Patient Characteristics

**Figure 1:**
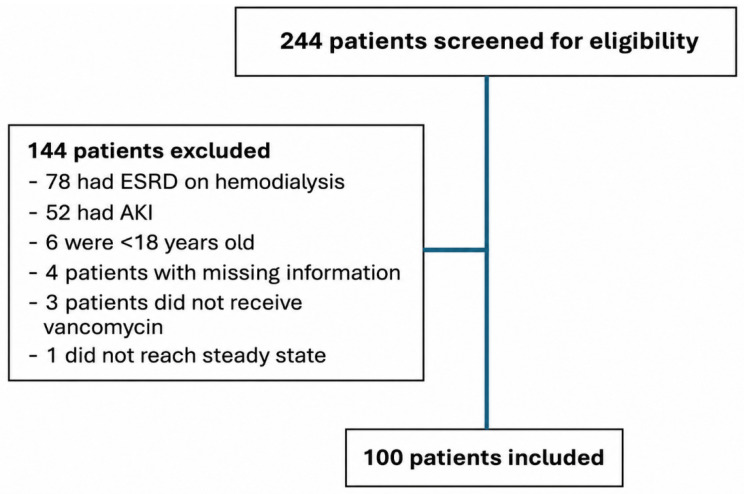
Flow chart of the study.

A total of 244 patients were screened for eligibility, and 100 were included in the study (Figure 1). Baseline demographic, clinical, and vancomycin therapy characteristics are summarized in Table 1. The mean age was 54.2 ± 20.0 years, and 53.0% were male. The median creatinine clearance was 72.0 mL/min [IQR 45.0–112.8], and the median SOFA score was 4.0 [IQR 2.0–6.0]. Diabetes was present in 44.0%, and cardiovascular disease in 52.0%; chronic kidney disease was uncommon (4.0%) per the inclusion criteria. The median vancomycin maintenance dose was 1000 mg [IQR 887.5–1000], with a median dosing interval of 12 h, yielding a median total daily dose of 27.8 mg/kg/day [IQR 20.2–33.6]. A loading dose was administered to 24.0% of patients. 18 patients (18%) were receiving a concomitant nephrotoxin at the time of vancomycin initiation. 5 patients (5%) were on acyclovir, and 13 patients (13%) were on piperacillin/tazobactam.

Among the 100 critically ill patients included in the study, 34 (34.0%) had microbiologically confirmed infections. Bloodstream infections were the most common source of infection (13/34, 38.2%), followed by respiratory infections (8/34, 23.5%), wound infections (2/34, 5.9%), central nervous system infections (2/34, 5.9%), and other sources, including ear swab, urine, tracheal aspirate, sterile body fluid aspirate, and smear specimens. Mixed infections involving more than one pathogen were identified in several patients. The most commonly isolated pathogens in the patient population were *Staphylococcus aureus* (including methicillin-resistant *S. aureus* [MRSA]), *Enterococcus faecalis*, *Enterococcus faecium*, *Pseudomonas aeruginosa*, *Klebsiella pneumoniae*, *Acinetobacter baumannii*, and coagulase-negative staphylococci (*Staphylococcus epidermidis*, *S. capitis*, *S. haemolyticus*, and *S. hominis*). Other less commonly isolated organisms included *Escherichia coli*, *Morganella morganii*, *Streptococcus anginosus*, *Streptococcus mitis*, *Gemella morbillorum*, *Serratia marcescens*, and *Candida* species.

### 2.2. Trough and AUC_0–24_/MIC Distributions

The mean steady-state trough concentration at the first measurement was 14.8 ± 8.0 mg/L, and the mean ClinCalc-derived AUC_0–24_/MIC was 499.7 ± 201.3 mg·h/L. By trough category, 59 patients (59.0%) were below 15 mg/L, 20 (20.0%) were within the 15–20 mg/L target band, and 21 (21.0%) exceeded 20 mg/L. By AUC_0–24_/MIC category, 22 (22.0%) were subtherapeutic (<400), 67 (67.0%) were on-target (400–600), and 11 (11.0%) were supratherapeutic (>600 mg·h/L). Trough on-target attainment using the institutional 15–20 mg/L target was therefore 20.0%, whereas AUC_0–24_/MIC on-target attainment was 67.0%.

### 2.3. Discordance Between Trough and AUC_0–24_/MIC Classifications

**Figure 2:**
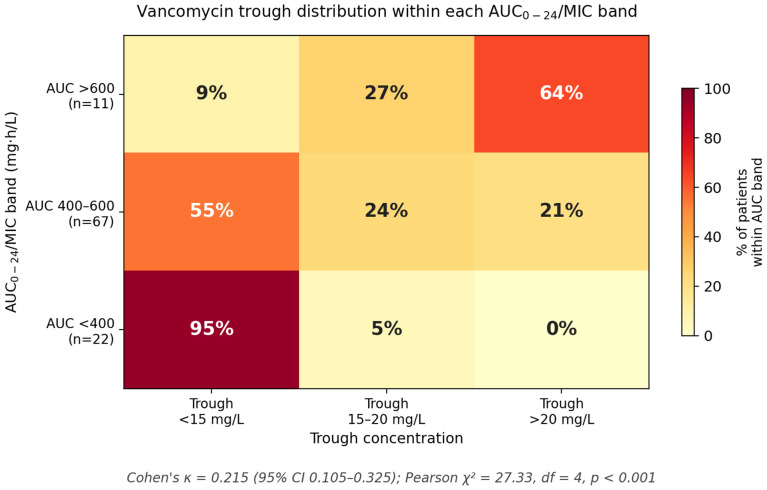
Vancomycin trough concentration distribution within each AUC_0–24_/MIC band.

Table 2 presents the full cross-tabulation of trough and AUC_0–24_/MIC categories, with the visualization shown in Figure 2. The two classifications are significantly correlated (Pearson χ^2^ = 27.33, df = 4, *p* < 0.001), but they only demonstrate fair agreement in the 3 × 3 ordinal classification (Cohen’s κ = 0.215, 95% CI 0.105–0.325). The Bowker test showed that the pattern of disagreement was directionally biased rather than random (χ^2^ = 42.22, df = 3, *p* < 0.001), primarily indicating trough underestimation of AUC_0–24_/MIC exposure. When classifying patients as either clinically actionable on-target (trough 15–20 mg/L; AUC_0–24_/MIC 400–600 mg·h/L) or off-target, the two methods disagreed in 55 of 100 patients (55.0%, 95% CI, 45.2–64.4). This highlights that the dichotomous, trough-based dose-adjustment decision differs from the AUC-based decision in more than half of cases.

#### Discordance Scenarios

**Figure 3:**
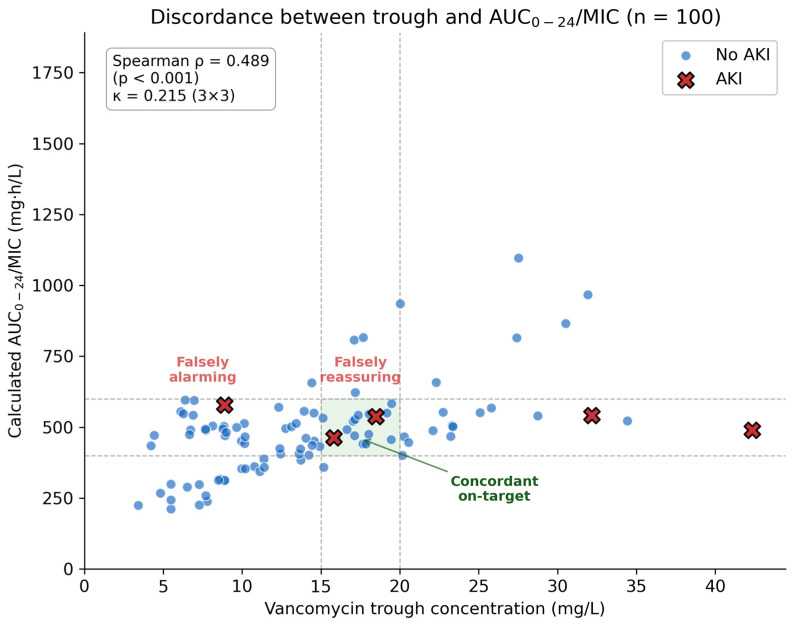
Scatterplot of trough concentration versus calculated AUC_0–24_/MIC Vertical reference lines indicate trough cutoffs at 15 and 20 mg/L; horizontal reference lines indicate AUC_0–24_/MIC cutoffs at 400 and 600 mg·h/L. The shaded green region marks the concordant on-target zone; AKI events (*n* = 5) are highlighted as red crosses. Spearman ρ = 0.489 (*p* < 0.001).

Table 3 details the two predefined discordance scenarios, and Figure 3 shows them. The “falsely alarming” scenario—where trough levels are below 15 mg/L despite an AUC_0–24_/MIC of ≥400 mg·h/L—occurred in 38 patients (38.0%, 95% CI 29.1–47.8). This suggests that strictly following a dose-escalation strategy aimed at trough targets could lead to unnecessary dose increases in more than a third of patients who already have adequate vancomycin exposure. Conversely, the “falsely reassuring” scenario occurred in 3 patients (3.0%), all of whom had trough levels between 15 and 20 mg/L but AUCs exceeding 600 mg·h/L. These patients may face an increased risk of nephrotoxicity that is not detected by trough monitoring alone.

### 2.4. Probability of Supratherapeutic AUC_0–24_/MIC by Trough Band

The percentage of patients with supratherapeutic AUC_0–24_/MIC (>600 mg·h/L) increased consistently across trough levels: 1.7% in those with troughs under 15 mg/L, 15.0% in the 15–20 mg/L range, and 33.3% in those with troughs above 20 mg/L. The unadjusted odds ratio for having a supratherapeutic AUC_0–24_/MIC in patients with troughs of 15–20 mg/L compared to those with troughs less than 15 mg/L was 10.24 (95% CI 1.00–104.87; Fisher exact *p* = 0.048), indicating a significant link at the higher end of the usual therapeutic trough levels. No patient with a trough below 10 mg/L had an AUC_0–24_/MIC above 600 mg·h/L.

### 2.5. Predictors of AUC_0–24_/MIC

In univariate analysis, only trough was significantly correlated with AUC_0–24_/MIC (Spearman ρ = 0.489, *p* < 0.001). Variables such as CrCl, BMI, total daily dose, age, weight, and SOFA score did not show significant associations. The multiple linear regression results are summarized in Table 4: trough was the only independent predictor of AUC_0–24_/MIC (β = 15.50 mg·h/L per 1 mg/L increase in trough; 95% CI, 11.21 to 19.80; *p* < 0.001). The model had an R^2^ of 0.373 (adjusted R^2^ = 0.347) and an overall F statistic of 14.12 (*p* < 0.001). No significant multicollinearity was identified among the included variables.

### 2.6. Acute Kidney Injury

**Figure 4:**
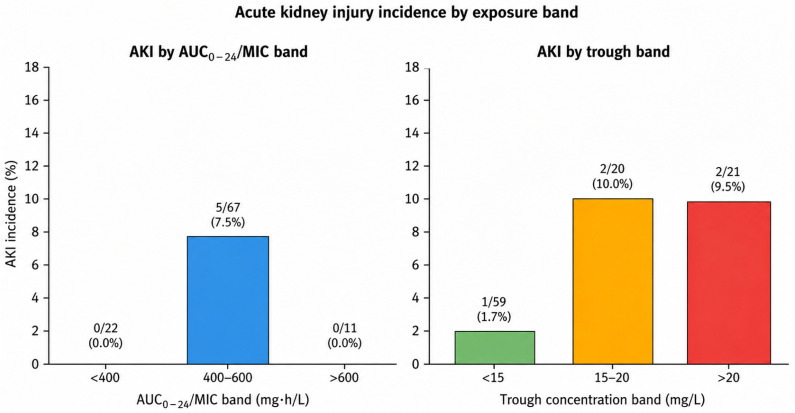
Acute kidney injury incidence by AUC_0–24_/MIC band and by trough concentration band.

Within 48 h of starting vancomycin, AKI was observed in 5 patients (5.0%), including 4 who met stage 1 criteria and 1 who met stage 2 criteria. By AUC, the incidence was 0/22 (0.0%) for AUC_0–24_/MIC < 400, 5/67 (7.5%) for AUC_0–24_/MIC 400–600, and 0/11 (0.0%) for AUC_0–24_/MIC > 600 mg·h/L (Pearson χ^2^ = 2.59, *p* = 0.274). By trough levels, the incidence was 1/59 (1.7%) for trough < 15, 2/20 (10.0%) for trough 15–20, and 2/21 (9.5%) for trough > 20 mg/L (Pearson χ^2^ = 3.31, *p* = 0.191) (Figure 4).

Among the five AKI cases, baseline creatinine clearance ranged widely from 5 to 149 mL/min, and SOFA scores ranged from 5 to 9. One patient received concomitant piperacillin/tazobactam, and four had no documented exposure to nephrotoxins. No consistent clinical characteristics distinguished patients who developed AKI.

Exploratory diagnostic characteristics of predefined trough and AUC_0–24_/MIC thresholds for identifying AKI are shown in Table 5: sensitivity was highest for trough > 15 mg/L (80.0%), moderate for AUC_0–24_/MIC > 500 mg·h/L (60.0%) and trough > 20 mg/L (40.0%), and zero for AUC_0–24_/MIC > 600 mg·h/L. Fisher exact tests comparing AKI rates between high- and low-exposure groups were not statistically significant (trough > 20 vs. ≤20: *p* = 0.28; AUC_0–24_/MIC > 600 vs. ≤600: *p* = 1.00), likely due to the small number of events.

## 3. Discussion

Our study aimed to compare Bayesian calculator-derived AUC_0–24_/MIC with trough-level vancomycin monitoring in critically ill patients. In our single-center retrospective study of 100 critically ill ICU patients, we confirmed significant discordance between trough-based vancomycin monitoring and single-sample Bayesian calculator-derived AUC_0–24_/MIC. Whereas only 20% of patients achieved the trough level target (15–20 mg/L), 67% of participants achieved the AUC_0–24_/MIC target (400–600 mg·h/L). The results showed a significant association with AUC_0–24_/MIC, and the overall agreement between the two methods was moderate. Notably, a large proportion of patients were subtherapeutic by trough levels despite achieving the target AUC_0–24_/MIC range. This was supported by a prospective observational study of 47 patients, in which the discordance between trough and AUC_0–24_/MIC was 52.9%. Most patients had trough levels < 15 mg/L and AUCs > 400 mg·h/L [9]. Another trial by Alzahrani and his team found that the target AUC_0–24_ (400 to 600 mg·h/L) and measured trough (10 to 20 mg/L) were documented in 127 (37.1%) and 185 (54%) cases, respectively [4]. Additionally, a study by Hale et al. showed that only 35 patients (45.7%) had trough levels of 15–20 mg/L and an AUC_0–24_/MIC ≥ 400 mg·h/L [10].

When calculating the AUC_0–24_ using Bayesian modeling, it is recommended to obtain two samples: one at peak (2 h post-infusion) and another at trough (30–60 min before the next dose). While a trough concentration alone may be sufficient to predict the AUC_0–24_/via the Bayesian method, as supported by the revised therapeutic drug monitoring guideline [2]. In addition, obtaining two blood samples for each vancomycin monitoring is difficult, especially in high-workload environments like the ICU, and it increases institutional costs and the risk of out-of-stock monitoring resources. A nationwide cross-sectional survey conducted in China found that around 59.5% (78 out of 131) of the surveyed hospitals conducted vancomycin therapeutic drug monitoring (TDM); however, only 10.7% (14 out of 131) of these institutions implemented AUC-based vancomycin TDM, and only 37 institutions had the ability to estimate the AUC_0–24_/MIC (37 out of 161, 23.0%). According to the national survey, the low implementation rate was due to several factors, including the high cost of AUC-based monitoring; inadequate knowledge among pharmacists and/or physicians; the complexity of AUC_0–24_/MIC calculations; difficulty obtaining AUC_0–24_/MIC software; and unclear benefit of AUC-based monitoring [11]. Alternatively, using a validated single-sample Bayesian calculator-derived AUC_0–24_/MIC can provide an AUC_0–24_/MIC in a much easier way for vancomycin monitoring, thereby improving therapeutic precision, avoiding unnecessary dose escalation, and optimizing maximum benefit with vancomycin. This implementation was supported by Olney et al., who found a strong correlation observed between Bayesian two-level and one-level methods (r = 0.931), with an overall 88.5% clinical decision agreement [12].

In a comparison between single-sample Bayesian AUC estimation and two-sample approaches, the 2020 ASHP/IDSA/PIDS/SIDP guideline states that Bayesian AUC can be estimated using one or two concentrations, with a preference for two-point concentrations, as this approach provides the highest possible precision and validation when estimating a patient’s individual AUC. Recent evidence supports this recommendation. Dreyse et al. assessed Bayesian vancomycin AUC estimation in critically ill patients and found that two-level sampling yields more precise, less biased AUC/MIC estimates than single-level methods (*p* = 0.042) [13]. Similarly, Olney et al. compared Bayesian methods using one or two concentrations in hospitalized adults. Although clinical decisions generally showed high agreement, they found that one-level Bayesian estimates could differ from those of two-level methods. Specifically, when the Bayesian two-concentration AUC was classified as supratherapeutic, the one-concentration estimate was reclassified as therapeutic or subtherapeutic, with proportions of 14.7% and 1.0%, respectively. Conversely, when the AUC_24_ was deemed subtherapeutic by the two-concentration method, the one-concentration approach predicted a therapeutic AUC24 in 12.8% of such cases [14]. Moreover, in one study by Ondrush et al., vancomycin AUC was compared between two-point pharmacokinetics and single-concentration estimates using two online vancomycin calculators. They found that AUC24 estimates from a single concentration showed some bias and imprecision compared with the two-concentration method [14]. Nevertheless, a study by Khanh Vy demonstrated that Bayesian one-concentration methods generally correlate well with two-concentration approaches, which showed high agreement between the Bayesian two-concentration and one-concentration methods (r = 0.974; clinical agreement, 91%) [15], but it does not rule out misclassification between the two methods. Therefore, although single-sample Bayesian estimation provides a practical solution for institutions with limited two-level monitoring, it may lead to some exposure misclassification, especially in ICU patients experiencing augmented renal clearance, fluid shifts, vasopressor use, or rapid changes in renal function. Accordingly, our findings should be interpreted as a real-world assessment of a pragmatic single-sample approach rather than a validation against a two-concentration reference standard.

One important finding is the direction of the discordance. In our study, the dominant pattern was “falsely alarming,” observed in 38 patients. This pattern can lead to unnecessary dose escalation in high-risk ICU patients to AKI, exposing them to avoidable cumulative nephrotoxin exposure without an expected efficacy benefit. In contrast, in one prior report on non-ICU patients, the predominant pattern was “falsely reassuring,” observed in 69 patients. Although the absolute number of “falsely reassuring” patients within our cohort was minimal (*n* = 3), the observed pattern replicates the safety signal described by Alzahrani and colleagues [4]. Furthermore, it substantiates the 2020 guideline’s prudence in noting that trough levels of 15–20 mg/L may be associated with potentially nephrotoxic AUC_0–24_/MIC exposure [2,4]. The difference can be explained by the fact that critically ill patients exhibit pharmacokinetic alterations, such as augmented renal function, hemodynamic instability, organ support, and an expanded volume of distribution due to fluid resuscitation, compared with non-ICU patients admitted to general wards [16].

The secondary AKI analysis was considered exploratory, and the results should be interpreted cautiously, given the low number of events (5 AKI events among 100 patients). Unexpectedly, all AKI events occurred in patients with AUC_0–24_/MIC values within the guideline-recommended range (400–600 mg·h/L), whereas no events were observed in the supratherapeutic AUC group (>600 mg·h/L).

Within these constraints, two notable patterns emerged. First, the traditional trough threshold of >20 mg/L identified only 40% of AKI cases (2 of 5 events), whereas a more sensitive trough threshold of >15 mg/L detected 80% (4 of 5 events), albeit with a corresponding false-positive rate of 39%. A study comparing AUC-guided monitoring with traditional trough-guided monitoring found that the rate of AKI was higher with trough monitoring (20%) than with AUC/MIC monitoring (18%); however, the difference was not statistically significant [17]. Second, no AKI events occurred within the >600 mg·h/L category, as all five events occurred within the on-target AUC_0–24_/MIC range of 400–600. These findings may reflect the multifactorial nature of AKI in critically ill patients, in which factors such as hemodynamic instability, concomitant nephrotoxins, or dehydration may contribute independently of vancomycin exposure. Previous studies have demonstrated an association between higher vancomycin exposure and increased AKI risk. Niwa et al. showed that AUC, calculated from a single trough concentration, was a more accurate predictor of nephrotoxicity than trough concentration [18]. In one cohort, 68.9% reached an AUC_0–24_/MIC of 400–600 mg·h/L, and 24.2% had supratherapeutic levels (>600 mg·h/L). Patients with AKI had higher vancomycin troughs (22.1 ± 9.9 vs. 13.8 ± 3.6 mg/L, *p* < 0.001) and AUCs (695.9 ± 236.6 vs. 576.4 ± 94.2 mg·h/L, *p* < 0.001). The proportion with AUC/MIC ≥400 was similar across groups, indicating that nephrotoxicity is more related to excessive exposure than to therapeutic targets, which contradicts our study’s findings [18].

The discrepancy between our findings and previous studies is likely due to the very small number of AKI cases, the 48-h assessment period, and the complex causes of AKI in critically ill patients. In our cohort, AKI cases varied widely in baseline kidney function (creatinine clearance from 5 to 149 mL/min) and illness severity (SOFA score 5–9), with one patient also on piperacillin/tazobactam, a drug known to be nephrotoxic. Other factors we did not measure, such as hemodynamic instability, sepsis severity, fluid balance, and exposure to additional nephrotoxins, may have also played a role in kidney injury. Consequently, the lack of AKI in patients with AUC_0–24_/MIC > 600 mg·h/L does not be interpreted as evidence that high vancomycin exposure is safe; rather, it reflects the limited number of events and possible confounding factors in this retrospective analysis.

From a clinical patient care perspective, our data indicate that dependence on trough-only monitoring may lead to unnecessary vancomycin dose escalation in critically ill patients, despite sufficient overall drug exposure. This has significant implications for patient safety, especially in ICU populations, who are at greater risk of nephrotoxicity. We employed trough level measurements rather than a two-sample approach because the majority of institutions did not adopt AUC monitoring or the two-sample approach for vancomycin monitoring. Additionally, single-sample Bayesian AUC_0–24_/MIC calculation may offer a practical and resource-efficient alternative for institutions that rely solely on trough monitoring. This technique may promote wider implementation of guideline-recommended AUC-guided monitoring in real-world ICU practice by reducing reliance on trough levels alone for vancomycin monitoring, aligning with the 2020 ASHP/IDSA guideline for monitoring vancomycin [2].

To the best of our knowledge, this is the first study to provide a comprehensive comparison of Bayesian calculator-derived AUC_0–24_/MIC and trough levels in critically ill patients. However, our study has several limitations. First, the retrospective design and reliance on electronic health record extraction limit our ability to capture all relevant covariates and introduce selection bias; however, we mitigated this by predefining inclusion and exclusion criteria and using a standardized data collection form. Second, the AUC_0–24_/MIC was estimated from a single trough sample using the Bayesian module of an open-access calculator rather than a two-sample reference standard. Nevertheless, the 2020 ASHP/IDSA guideline endorses single-concentration Bayesian estimation as acceptable but notes that two-sample fitting yields higher precision, particularly in critically ill patients. Third, all AUC/MIC calculations assumed an MIC of 1 mg/L. Organism-specific MIC values were not routinely documented in the electronic health records, and no pathogen was isolated in 66% of the cohort, making isolate-specific MIC adjustment infeasible. Fourth, the risk of AKI after 48 h was not captured; other AKI causes, such as hemodynamic instability and intravenous contrast administration, were not collected. All five AKI events occurred within the AUC_0–24_/MIC 400–600 mg·h/L category, so supratherapeutic AUC_0–24_/MIC (>600 mg·h/L) did not identify any AKI in this cohort. Our multivariate analysis was limited, potentially underestimating the significance of supratherapeutic levels or overfitting due to the small sample size. This finding should be interpreted with caution because of the few AKI events, the short follow-up, and the exploratory nature of the study. Further studies with larger sample sizes are needed to validate these safety findings. Additionally, the primary ICU admission diagnosis was not collected because it fell outside the scope of this pharmacokinetic and therapeutic drug monitoring study. As a result, we could not assess whether the underlying critical illness affected the discordance between trough- and AUC-based target achievement. Lastly, this is a single-center study, which may limit its generalizability.

## 4. Methods

### 4.1. Study Design and Setting

This retrospective, single-center cohort study was conducted in the intensive care units (ICUs) at Almoosa Specialist Hospital in Al-Ahsa, Saudi Arabia, from January 2024 to December 2025. Demographic, clinical, dosing, and laboratory data were extracted from the electronic health record using a standardized data collection form. Almoosa Specialist Hospital was selected for its advanced ICU settings, expertise in managing critically ill patients, accessible electronic records, and willingness to participate.

### 4.2. Participants

Patients aged ≥ 18 years who were admitted to the ICU, received vancomycin, and had a steady-state vancomycin trough level were screened for eligibility. We excluded patients aged < 18 years, those with unstable kidney function, those on hemodialysis (conventional or peritoneal) or continuous renal replacement therapy, those with acute kidney injury before vancomycin administration, and those with missing information.

### 4.3. Vancomycin Dosing and Monitoring

Vancomycin was initiated at 15–20 mg/kg of actual body weight per dose, with the dosing interval (8, 12, or 24 h) determined by creatinine clearance calculated using the Cockcroft-Gault equation. A loading dose was administered at the prescribing clinician’s discretion. Steady-state trough concentrations were obtained 30 min before the fourth dose; subsequent levels were obtained as clinically indicated, based on dose adjustments or changes in renal function.

During the study period, therapeutic drug monitoring relied exclusively on trough concentrations, and vancomycin dose adjustments were made by clinical pharmacists in accordance with institutional practice. Bayesian AUC_0–24_/MIC monitoring was not routinely available and was not used to guide clinical management. For this study, AUC_0–24_/MIC values were calculated retrospectively from the same trough concentration using the ClinCalc online Bayesian calculator. These retrospective AUC calculations were performed solely for research purposes to compare target attainment between conventional trough-based monitoring and Bayesian-derived AUC monitoring; these calculations were not available to the treating physicians or clinical pharmacists and did not influence patient management.

### 4.4. AUC_0–24_/MIC Estimation

AUC_0–24_ was calculated for each trough sample using the freely available ClinCalc Vancomycin Calculator (clincalc.com) with the Bayesian estimation module selected [8]. The Bayesian module integrates patient-specific covariates (weight, age, gender, serum creatinine, CrCl) with a population pharmacokinetic prior to derive individualized posterior estimates of vancomycin clearance, volume of distribution, elimination rate constant, and the resulting 24-h AUC. The AUC_0–24_/MIC ratio was computed assuming a target organism MIC of 1 mg/L, consistent with the 2020 ASHP/IDSA consensus guideline when MIC is not directly measured [2].

### 4.5. Outcomes

The primary outcome of the study was the discordance in target attainment when classified by trough versus by AUC_0–24_/MIC. The secondary outcome was the incidence of acute kidney injury (AKI) within 48 h of vancomycin initiation.

### 4.6. Target Attainment Definitions

Trough on-target was defined as 15–20 mg/L (3); for stratified analyses, trough categories were <15, 15–20, and >20 mg/L. AUC_0–24_/MIC on-target was defined as 400–600 mg·h/L [2]; AUC_0–24_/MIC categories were <400 (sub-therapeutic), 400–600 (on-target), and >600 mg·h/L (supratherapeutic). Two clinically meaningful discordance scenarios were prespecified: (1) “falsely reassuring”—trough 15–20 mg/L with AUC_0–24_/MIC > 600 mg·h/L, in which conventional trough-guided practice would consider exposure adequate, even though the patient is in the supratherapeutic range; and (2) “falsely alarming”—trough < 15 mg/L with AUC_0–24_/MIC ≥ 400 mg·h/L, in which conventional trough-guided practice would prompt dose escalation despite already-adequate exposure.

### 4.7. Acute Kidney Injury (AKI)

AKI was defined according to the Kidney Disease Improving Global Outcomes (KDIGO) criteria [19]: an absolute increase in serum creatinine of ≥26.5 μmol/L (≥0.3 mg/dL) or a relative increase of ≥1.5× baseline within 48 h of vancomycin initiation. KDIGO stage was assigned as stage 1 (≥26.5 μmol/L absolute or 1.5–1.9× baseline), stage 2 (2.0–2.9× baseline), or stage 3 (≥3.0× baseline or absolute SCr ≥ 353.6 μmol/L).

### 4.8. Statistical Analysis

Continuous variables were summarized as mean ± SD for normally distributed data and as median [IQR] for non-normal data. Categorical variables were reported as frequencies and percentages. Normality was assessed using the Shapiro–Wilk test and visual inspection. Trough concentrations and AUC_0–24_/MIC values were also presented as mean ± SD to facilitate clinical interpretation and comparison with prior pharmacokinetic studies. Available-case analysis was used for variables with SOFA available in 95 patients, CRP in 97, procalcitonin in 85, and all other variables in all 100 patients.

Discordance between trough and AUC_0–24_/MIC classifications was assessed using a 3 × 3 cross-tabulation. Agreement was quantified with Cohen’s κ. To explore associations and patterns of disagreement between trough and AUC_0–24_/MIC categories, Pearson χ^2^ and Bowker tests were used. Wilson 95% confidence intervals were computed for binomial proportions. The relationship between trough categories and supratherapeutic AUC_0–24_/MIC exposure (>600 mg·h/L) was examined using Pearson χ^2^, Fisher’s exact test, and odds ratios. Also, Spearman correlation analysis was conducted to evaluate associations between trough concentration and AUC_0–24_/MIC.

Multiple linear regression was used to identify predictors of AUC_0–24_/MIC, including trough concentration, creatinine clearance, BMI, and total daily dose. In the Supplementary Materials (Table S1), an exploratory multinomial logistic regression was used to identify factors associated with subtherapeutic and supratherapeutic AUC_0–24_/MIC categories, with the on-target AUC_0–24_/MIC group (400–600 mg·h/L) as the reference category. Moreover, sensitivity analyses using binary logistic regression were conducted for supratherapeutic and off-target AUC_0–24_/MIC classifications.

The exploratory diagnostic performance of selected trough and AUC_0–24_/MIC thresholds for predicting AKI was evaluated using sensitivity, specificity, positive predictive value, and negative predictive value. All tests were two-sided, and *p* < 0.05 was considered statistically significant. All statistical analyses were conducted using IBM SPSS Statistics version 31.0.1.0.

## 5. Conclusions

In critically ill ICU patients, the calculator-derived AUC_0–24_/MIC showed significant discordance with single-trough vancomycin monitoring, primarily indicating under-exposure by trough criteria in patients with adequate AUC_0–24_/MIC levels. This discrepancy could lead to unnecessary dose increases. More clinical trials with larger sample sizes are needed to confirm this finding in critically ill patients.

## Figures and Tables

**Table 1:** Baseline characteristics of the study cohort (*n* = 100).

Variable	Value (*n* = 100)
**Demographics**	
Age, years, mean ± SD	54.2 ± 20.0
Sex, male, *n* (%)	53 (53.0)
Weight, kg, median [IQR]	73.0 [58.2–90.2]
Height, cm, mean ± SD	161.6 ± 10.5
BMI, kg/m^2^, median [IQR]	27.2 [23.9–34.0]
**Renal function & severity**	
CrCl (Cockcroft-Gault), mL/min, median [IQR]	72.0 [45.0–112.8]
Baseline serum creatinine, μmol/L, median [IQR]	70.0 [52.0–82.5]
SOFA score, median [IQR]	4.0 [2.0–6.0]
Initial lactate, mmol/L, median [IQR]	1.3 [0.9–1.6]
MAP at admission, mmHg, median [IQR]	86.0 [76.8–97.0]
Temperature, °C, median [IQR]	36.8 [36.5–37.3]
**Inflammatory markers**	
CRP, mg/L, median [IQR]	95.4 [43.2–170.5]
Procalcitonin, ng/mL, median [IQR]	0.2 [0.1–1.0]
**Comorbidities, *n* (%)**	
Diabetes mellitus	44 (44.0)
Cardiovascular disease	52 (52.0)
Respiratory failure	38 (38.0)
Malignancy	12 (12.0)
Chronic kidney disease	4 (4.0)
Pathogen documented	34 (34.0)
**Vancomycin therapy**	
Loading dose administered, *n* (%)	24 (24.0)
Maintenance dose, mg, median [IQR]	1000 [887.5–1000]
Dosing interval, h, median [IQR]	12.0 [12.0–12.0]
Total daily dose, mg/day, median [IQR]	2000 [2000–2250]
Total daily dose, mg/kg/day, median [IQR]	27.8 [20.2–33.6]
Concomitant nephrotoxin, *n* (%)	18 (18%)
**Therapeutic drug monitoring**	
First trough, mg/L, mean ± SD ^†^	14.8 ± 8.0
First AUC_0–24_/MIC, mg·h/L, mean ± SD ^†^	499.7 ± 201.3
Trough on-target (15–20 mg/L), *n* (%)	20 (20.0)
AUC_0–24_/MIC on-target (400–600 mg·h/L), *n* (%)	67 (67.0)

BMI, body mass index; CrCl, creatinine clearance (Cockcroft-Gault); SOFA, Sequential Organ Failure Assessment; CRP, C-reactive protein; MAP, mean arterial pressure; AUC, area under the concentration–time curve; ^†^ medians [IQR] trough and AUC_0–24_/MIC were 13.7 [8.8–18.1] mg/L and 485 [406–544] mg·h/L, respectively.

**Table 2:** Patients’ cross-tabulation of trough and AUC_0–24_/MIC categories (*n* = 100).

Trough Band (mg/L)	AUC_0–24_/MIC < 400	AUC_0–24_/MIC 400–600	AUC_0–24_/MIC > 600	Total	% AUC_0–24_/MIC > 600
**<15**	21	37	1	59	1.7%
**15–20**	1	16	3	20	15.0%
**>20**	0	14	7	21	33.3%
**Total**	22	67	11	100	11.0%

Diagonal cells (trough < 15/AUC_0–24_/MIC < 400; trough 15–20/AUC_0–24_/MIC 400–600; trough > 20/AUC_0–24_/MIC > 600) represent classifications that agree between the two methods; off-diagonal cells represent discordance. Cohen’s κ = 0.215 (95% CI 0.105–0.325). Pearson χ^2^ = 27.33, df = 4, *p* < 0.001. Bowker test of symmetry χ^2^ = 42.22, df = 3, *p* < 0.001.

**Table 3:** Discordance scenarios between trough and AUC_0–24_/MIC classification (*n* = 100).

Discordance Scenario	*n*	%	95% CI
**Concordant on-target (trough 15–20 AND AUC_0–24_/MIC 400–600)**	16	16.0	10.1–24.4
**Falsely reassuring (trough 15–20 mg/L but AUC_0–24_/MIC > 600 mg·h/L)**	3	3.0	1.0–8.5
**Falsely alarming (trough < 15 mg/L but AUC_0–24_/MIC ≥ 400 mg·h/L)**	38	38.0	29.1–47.8
**Overall binary discordance (on-target classifications disagree)**	55	55.0	45.2–64.4

**Table 4:** Multiple linear regression predicting AUC_0–24_/MIC (*n* = 100).

Predictor	β	SE	95% CI	*p*-Value
**Intercept**	264.14	78.25	108.79 to 419.50	0.001
**Trough (mg/L)**	15.50	2.16	11.21 to 19.80	<0.001
**CrCl (mL/min)**	0.10	0.43	–0.75 to 0.95	0.820
**BMI (kg/m^2^)**	0.27	1.80	–3.31 to 3.84	0.882
**TDD (mg/day)**	–0.004	0.027	–0.059 to 0.050	0.871

CrCl, creatinine clearance; BMI, body mass index; TDD, total daily dose.

**Table 5:** Exploratory diagnostic characteristics of predefined vancomycin exposure thresholds for AKI.

Threshold	TP	FP	FN	TN	Sensitivity	Specificity
**Trough > 20 mg/L**	2	19	3	76	40.0%	80.0%
**Trough > 15 mg/L**	4	37	1	58	80.0%	61.1%
**AUC_0–24_/MIC > 600 mg·h/L**	0	11	5	84	0.0%	88.4%
**AUC_0–24_/MIC > 500 mg·h/L**	3	38	2	57	60.0%	60.0%

TP, true positive; FP, false positive; FN, false negative; TN, true negative.

## Data Availability

The data presented in this study are available on request from the corresponding author due to institutional privacy regulations and ethical restrictions and are subject to approval by the institutional review board.

## Supplementary Materials

The following supporting information can be downloaded at: https://www.mdpi.com/article/10.3390/ph19071109/s1. 1. Exploratory Multinomial Logistic Regression Analysis. Table S1: Multinomial logistic regression for AUC_0–24_/MIC group (reference: on-target 400–600 mg·h/L). 2. Sensitivity analysis.

## Abbreviations

AKI	Acute Kidney Injury
AUC_0–24_/MIC	24-Hour Area Under the Curve to Minimum Inhibitory Concentration Ratio
ASHP	American Society of Health-System Pharmacists
BMI	Body Mass Index
ESRD	End-stage renal disease
CI	Confidence Interval
CrCl	Creatinine Clearance
CRP	C-Reactive Protein
EHR	Electronic Health Record
FP	False Positive
FN	False Negative
TN	True Negative
TP	True Positive
ICU	Intensive Care Unit
IDSA	Infectious Diseases Society of America
IQR	Interquartile Range
KDIGO	Kidney Disease: Improving Global Outcomes
MAP	Mean Arterial Pressure
MIC	Minimum Inhibitory Concentration
MRSA	Methicillin-Resistant *Staphylococcus aureus*
OR	Odds Ratio
PCR	Polymerase Chain Reaction
PIDS	Pediatric Infectious Diseases Society
PK/PD	Pharmacokinetic/Pharmacodynamic
RRT	Renal Replacement Therapy
SD	Standard Deviation
SIDP	Society of Infectious Diseases Pharmacists
SOFA	Sequential Organ Failure Assessment
SCr	Serum Creatinine
TDD	Total Daily Dose
TDM	Therapeutic Drug Monitoring
WBC	White Blood Cell
