# Supplementary material for: Real-World Comparison of Trough- and Online-Bayesian-Calculator-Derived AUC0–24/MIC Target Attainment for Vancomycin Monitoring in Critically Ill Patients
Source: Pharmaceuticals (Basel). 2026 Jul 18;19(7):1109. doi: 10.3390/ph19071109 (PMC13414881; doi:10.3390/ph19071109)
Supplement: Supplementary file 1 [file pharmaceuticals-19-01109-s001.zip › pharmaceuticals-4439865-supplementary.pdf]

1. Exploratory Multinomial Logistic Regression Analysis:

In the multinomial logistic regression model, using the on-target $AUC_{0–24}$/MIC band as the reference, each 1 mg/L increase in trough was independently associated with a 21% decrease in the odds of falling into the sub-therapeutic $AUC_{0–24}$/MIC group (adj. OR 0.79, 95% CI 0.68–0.92, $p = 0.002$) and a 13% increase in the odds of being in the supra-therapeutic $AUC_{0–24}$/MIC group (adj. OR 1.13, 95% CI 1.04–1.24, $p = 0.004$). CrCl, BMI, and TDD were not statistically significant after adjustment in either comparison (Table S1). The overall model was significant (likelihood ratio $\chi^2 = 43.14$, df = 8, $p < 0.001$), with a 72.0% correct classification rate. The likelihood ratio test confirms trough's substantial independent contribution to the model ($\chi^2 = 29.19$, df = 2, $p < 0.001$).

Table S1. Multinomial logistic regression for $AUC_{0–24}$/MIC group (reference: on-target 400–600 mg·h/L).

Comparison	Predictor	β	Adj. OR	95% CI	p
**Low vs Normal**	Trough (per 1 mg/L)	–0.240	0.79	0.68–0.92	0.002
**Low vs Normal**	CrCl (per 1 mL/min)	0.004	1.00	0.99–1.02	0.630
**Low vs Normal**	BMI (per 1 kg/m²)	0.008	1.01	0.94–1.09	0.834
**Low vs Normal**	TDD (per 1 mg/day)	–0.001	1.00	1.00–1.00	0.082
**High vs Normal**	Trough (per 1 mg/L)	0.126	1.13	1.04–1.24	0.004
**High vs Normal**	CrCl (per 1 mL/min)	–0.012	0.99	0.97–1.01	0.239
**High vs Normal**	BMI (per 1 kg/m²)	0.005	1.01	0.94–1.08	0.890
**High vs Normal**	TDD (per 1 mg/day)	0.000	1.00	1.00–1.00	0.611

2. Sensitivity analysis

Because the supratherapeutic $AUC_{0–24}$/MIC (>600 mg·h/L) category included only 11 events, yielding about 2.75 events per predictor in the multinomial High-versus-Normal comparison, a pre-specified sensitivity analysis was performed. This used a binary logistic regression model

comparing supratherapeutic to non-supratherapeutic AUC, with the same four predictors. Trough concentration remained a strong, independent predictor of supratherapeutic AUC_{0-24}/MIC (adjusted OR 1.15 per 1 mg/L, 95% CI 1.06–1.25, $p = 0.001$; model omnibus $\chi^2 = 17.36$, df = 4, $p = 0.002$). The nearly identical adjusted ORs in both the binary and multinomial models (1.15 vs 1.13) suggest that the association between trough levels and supratherapeutic AUC_{0-24}/MIC is robust across models. Additionally, a second binary model comparing on-target (400–600) to off-target (below 400 or above 600) AUC_{0-24}/MIC showed no significant impact of trough (adjusted OR 1.02, 95% CI 0.96–1.08, $p = 0.620$). Overall, these findings mostly align with the main multinomial regression analysis.
